# Supplementary material for: No effect of additional education on long-term brain structure, a preregistered natural experiment in thousands of individuals
Source: eLife. 2025 Jul 25;13:RP101526. doi: 10.7554/eLife.101526 (PMC12296260; doi:10.7554/eLife.101526)
Supplement: Supplementary file 1. [file elife-101526-supp1.docx]

| Supplementary Table 1: Fuzzy RD Placebo Outcome results | | | | | | |
| --- | --- | --- | --- | --- | --- | --- |
| **Fuzzy RD Parameter** | **bandwidth** | **eff.obs** | **estimate** | **Confidence Interval** | **p.value** | **pFDR** |
| sex ~ EduAge16 \| running_var | 23.41 | 8008.29 | -0.28 | (-0.76, 0.20) | 0.266 | 0.384 |
| t2_FLAIR ~ EduAge16 \| running_var | 23.41 | 5112.74 | -0.28 | (-0.49, -0.07) | 0.009 | 0.059 |
| visit_day_correct ~ EduAge16 \| running_var | 27.44 | 9365.44 | 1023.63 | (123.00, 1924.25) | 0.025 | 0.083 |
| visit_day_correct^2^ ~ EduAge16 \| running_var | 25.61 | 8733.81 | 3675359.31 | (52771.30, 7297947.32) | 0.047 | 0.101 |
| headmotion ~ EduAge16 \| running_var | 27.05 | 6088.77 | 0.09 | (-0.10, 0.28) | 0.343 | 0.406 |
| summer ~ EduAge16 \| running_var | 20.14 | 6904.60 | -4.40 | (-5.64, -3.17) | 0.000 | 0.000^[[1]](#footnote-1)^ |
| imaging_center_11025 ~ EduAge16 \| running_var | 21.74 | 7380.04 | -0.56 | (-1.06, -0.07) | 0.025 | 0.083 |
| imaging_center_11026 ~ EduAge16 \| running_var | 25.18 | 8496.55 | 0.18 | (-0.15, 0.51) | 0.296 | 0.385 |
| imaging_center_11027 ~ EduAge16 \| running_var | 25.27 | 8527.40 | 0.27 | (-0.15, 0.70) | 0.215 | 0.350 |
| imaging_center_11028 ~ EduAge16 \| running_var | 23.11 | 7818.34 | 0.17 | (-0.06, 0.40) | 0.141 | 0.262 |
| dMRI_25922_1 ~ EduAge16 \| running_var | 27.06 | 5591.14 | 0.09 | (-0.50, 0.67) | 0.778 | 0.843 |
| dMRI_25921_1 ~ EduAge16 \| running_var | 27.03 | 5773.50 | 0.02 | (-0.54, 0.57) | 0.953 | 0.953 |
| dMRI_25928_1 ~ EduAge16 \| running_var | 26.28 | 5454.88 | 0.59 | (0.01, 1.16) | 0.046 | 0.101 |

1. Summer is a dummy coding variable for if the participants date of birth was in July or August. This makes it inherently weighed, *by design*, against ROSLA (which happened on September 1st). These children can be seen in Sup. Fig 2, see methods for further details. [↑](#footnote-ref-1)
