# Supplementary material for: No effect of additional education on long-term brain structure, a preregistered natural experiment in thousands of individuals
Source: eLife. 2025 Jul 25;13:RP101526. doi: 10.7554/eLife.101526 (PMC12296260; doi:10.7554/eLife.101526)
Supplement: Supplementary file 2. [file elife-101526-supp2.docx]

| Supplementary Table 2: Fuzzy RD Global Neuroimaging Results | | | | | | |
| --- | --- | --- | --- | --- | --- | --- |
| **Fuzzy RD Parameter** | **eff.obs** | **bandwidth** | **estimate (Y)** | **Confidence Interval** | **p.value** | **first.stage** |
| Surface Area | 7305.947 | 35.438 | 1055.733 | (-13654.9, 15766.37) | 0.889 | 0.084 |
| Cortical Thickness | 4606.983 | 22.242 | -0.107 | (-0.235, 0.02) | 0.099 | 0.099 |
| White Matter Hyperintensities | 4238.597 | 20.637 | 1440.618 | (-1835.03, 4716.27) | 0.395 | 0.101 |
| CSF normalized for head size | 4857.130 | 23.479 | 2989.622 | (-15306.03, 21285.27) | 0.753 | 0.097 |
| TBV normalized for head size | 5373.601 | 26.231 | 4602.620 | (-70961.58, 80166.82) | 0.906 | 0.095 |
| Mean Weighted FA | 4362.964 | 21.238 | -0.007 | (-0.02, 0.01) | 0.466 | 0.100 |
