# Supplementary material for: No effect of additional education on long-term brain structure, a preregistered natural experiment in thousands of individuals
Source: eLife. 2025 Jul 25;13:RP101526. doi: 10.7554/eLife.101526 (PMC12296260; doi:10.7554/eLife.101526)
Supplement: Supplementary file 3. [file elife-101526-supp3.docx]

| Supplementary Table 3: Fuzzy RD Uncorrected Global Neuroimaging Results | | | | | | |
| --- | --- | --- | --- | --- | --- | --- |
| **Fuzzy RD Parameter Uncorrected** | **eff.obs** | **bandwidth** | **estimate (uY)** | **Confidence Interval** | **p.value** | **first.stage** |
| Uncorrected Surface Area | 7939.988 | 38.722 | -5267.958 | (-23483.93, 12948.01) | 0.577 | 0.082 |
| Uncorrected Cortical Thickness | 4749.844 | 23.000 | -0.139 | (-0.275, -0.005) | 0.043 | 0.098 |
| Uncorrected White Matter Hyperintensities | 4467.847 | 21.778 | 2668.595 | (-856.54, 6193.73) | 0.141 | 0.099 |
| Uncorrected CSF normalized for head size | 4930.766 | 23.867 | 2968.307 | (-16016.26, 21952.87) | 0.764 | 0.097 |
| Uncorrected TBV normalized for head size | 5462.770 | 26.634 | -17635.849 | (-96743.57, 61471.87) | 0.667 | 0.094 |
| Uncorrected Mean Weighted FA | 4422.511 | 21.512 | -0.008 | (-0.03, 0.01) | 0.404 | 0.100 |
