# Supplementary material for: No effect of additional education on long-term brain structure, a preregistered natural experiment in thousands of individuals
Source: eLife. 2025 Jul 25;13:RP101526. doi: 10.7554/eLife.101526 (PMC12296260; doi:10.7554/eLife.101526)
Supplement: Supplementary file 4. [file elife-101526-supp4.docx]

|  | | Supplementary Table 4: One month window Bayesian Analysis | | | | | | | | |
| --- | --- | --- | --- | --- | --- | --- | --- | --- | --- | --- |
| **Y** | **X** | | **n** | **Estimate** | **Std Est.** | **95 Credible Interval** | **BF** | ***normal*(0, 0.5)** | ***normal*(0, 1.5)** |  |
| SA | ROSLA | | 232 | 5.72 | 0.00 | (-3144.86, 3131.35) | BF_01_=18.21 | BF_01_=9.22 | BF_01_=27.25 |  |
| SA | EduAge | | 229 | 833.24 | 0.14 | (248.86, 1438.26) | BF_10_=2.31 | BF_10_=4.71 | BF_10_=1.59 |  |
| CT | ROSLA | | 232 | 0.00 | -0.02 | (-0.03, 0.02) | BF_01_=15.09 | BF_01_=7.61 | BF_01_=22.48 |  |
| CT | EduAge | | 229 | 0.00 | -0.02 | (-0.01, 0.01) | BF_01_=14.38 | BF_01_=7.36 | BF_01_=21.33 |  |
| CSF_norm | ROSLA | | 232 | -664.54 | -0.04 | (-4828.25, 3446.43) | BF_01_=14.50 | BF_01_=7.34 | BF_01_=21.65 |  |
| CSF_norm | EduAge | | 229 | 775.82 | 0.12 | (-11.4, 1558.66) | BF_01_=2.33 | BF_01_=1.14 | BF_01_=3.58 |  |
| TBV_norm | ROSLA | | 232 | 3884.91 | 0.06 | (-12228.83, 20281.2) | BF_01_=13.61 | BF_01_=6.90 | BF_01_=20.30 |  |
| TBV_norm | EduAge | | 229 | -405.08 | -0.02 | (-3491.44, 2704.24) | BF_01_=14.47 | BF_01_=7.22 | BF_01_=21.74 |  |
| WM_hyper | ROSLA | | 232 | 222.81 | 0.07 | (-649.46, 1109.96) | BF_01_=13.26 | BF_01_=6.78 | BF_01_=20.04 |  |
| WM_hyper | EduAge | | 229 | -56.48 | -0.04 | (-224.85, 114.12) | BF_01_=12.04 | BF_01_=6.01 | BF_01_=17.93 |  |
| wFA | ROSLA | | 228 | 0.00 | -0.09 | (0, 0) | BF_01_=11.63 | BF_01_=6.00 | BF_01_=17.29 |  |
| wFA | EduAge | | 228 | 0.00 | 0.04 | (0, 0) | BF_01_=11.47 | BF_01_=5.78 | BF_01_=17.26 |  |

***Sup. Table 4 Caption****:* Only participants born in August and September 1957 are included. Bayesian analysis of global neuroimaging measures (Y) using a local randomization RD (“ROSLA”; dummy coding for participants born in September 1957) and the associational effect (EduAge) of the amount of attained education in years. The estimate is the median of the posterior reported in raw units. Std. Est is the standardized estimate (for EduAge this is a standardized continuous variable, while ROSLA is a dummy coded variable with 1 corresponding to being impacted by the policy). The estimate, CI & BF are reported for a normal prior (mean = 0, SD = 1). We also report Bayes factors (BF) for other priors. Two measures CSF & TBV are normalized for head size, this is reflected with “_norm”.
