# Supplementary material for: No effect of additional education on long-term brain structure, a preregistered natural experiment in thousands of individuals
Source: eLife. 2025 Jul 25;13:RP101526. doi: 10.7554/eLife.101526 (PMC12296260; doi:10.7554/eLife.101526)
Supplement: Supplementary file 5. [file elife-101526-supp5.docx]

| Supplementary Table 5: One Month window Test of Covariates | | | | | | |
| --- | --- | --- | --- | --- | --- | --- |
| **Y** | **X** | **n** | **Estimate** | **Bayes Factor** | **ci_low** | **ci_high** |
| sex | ROSLA | 394 | -0.04 | BF_01_=13.45 | -0.14 | 0.05 |
| visit_day_correct | ROSLA | 394 | 91.82 | BF_01_=12.17 | -91.10 | 274.77 |
| visit_day_correct^2^ | ROSLA | 394 | 320641.85 | BF_01_=14.03 | -429953.44 | 1075987.24 |
| headmotion | ROSLA | 260 | 0.01 | BF_01_=15.15 | -0.03 | 0.05 |
| imaging_center_11026 | ROSLA | 391 | 0.03 | BF_01_=14.51 | -0.04 | 0.10 |
| imaging_center_11027 | ROSLA | 391 | 0.04 | BF_01_=12.66 | -0.05 | 0.13 |
| dMRI_25922_1 | ROSLA | 232 | 0.02 | BF_01_=13.91 | -0.09 | 0.14 |
| dMRI_25921_1 | ROSLA | 239 | -0.12 | BF_01_=2.25 | -0.24 | 0.00 |
| dMRI_25928_1 | ROSLA | 233 | 0.05 | BF_01_=10.44 | -0.06 | 0.16 |
| imaging_center_11025 | ROSLA | 391 | -0.10 | BF_01_=2.42 | -0.20 | 0.00 |

***Sup. Table 5 Caption****:* A Bayesian local randomization analysis of placebo outcomes with participants born in August and September 1957 included. ROSLA dummy codes participants born in September 1957. Placebo outcomes are a common method to falsify an RD design, as seen above, by definition they *should* be unrelated to the natural experiment (ROSLA). The estimate is the median of the posterior. The estimate, CI & BF are reported for a normal prior (mean = 0, SD = 1).
