# Supplementary material for: No effect of additional education on long-term brain structure, a preregistered natural experiment in thousands of individuals
Source: eLife. 2025 Jul 25;13:RP101526. doi: 10.7554/eLife.101526 (PMC12296260; doi:10.7554/eLife.101526)
Supplement: Supplementary file 6. [file elife-101526-supp6.docx]

|  | | Supplementary Table 6: Five Month window Bayesian Analysis | | | | | | | |
| --- | --- | --- | --- | --- | --- | --- | --- | --- | --- |
| **Y** | **X** | | **n** | **Estimate** | **Std Est.** | **95 Credible Interval** | **BF** | ***normal*(0, 0.5)** | ***normal*(0, 1.5)** |
| SA | ROSLA | | 1195 | 901.66 | 0.06 | (-491.02, 2329.98) | BF_01_=9.17 | BF_01_=9.61 | BF_01_=28.59 |
| SA | EduAge | | 1185 | 594.31 | 0.10 | (305.26, 880.86) | BF_10_=41.70 | BF_10_=124.31 | BF_10_=55.39 |
| CT | ROSLA | | 1195 | -0.02 | -0.10 | (-0.02, 0) | BF_01_=7.22 | BF_01_=3.58 | BF_01_=11.00 |
| CT | EduAge | | 1185 | 0.00 | -0.05 | (-0.01, 0) | BF_01_=8.81 | BF_01_=4.48 | BF_01_=13.13 |
| CSF_norm | ROSLA | | 1203 | -788.23 | -0.05 | (-2524.2, 971.99) | BF_01_=24.18 | BF_01_=11.91 | BF_01_=36.13 |
| CSF_norm | EduAge | | 1193 | 761.90 | 0.12 | (409.83, 1123.57) | BF_10_=80.70 | BF_10_=381.73 | BF_10_=65.54 |
| TBV_norm | ROSLA | | 1203 | 3670.20 | 0.06 | (-3146.49, 10523.16) | BF_01_=20.28 | BF_01_=10.22 | BF_01_=30.81 |
| TBV_norm | EduAge | | 1193 | -1476.60 | -0.06 | (-2881.9, -68.34) | BF_01_=4.21 | BF_01_=2.11 | BF_01_=6.22 |
| WM_hyper | ROSLA | | 1193 | 2.07 | 0.00 | (-492.03, 501.21) | BF_01_=34.68 | BF_01_=17.62 | BF_01_=52.25 |
| WM_hyper | EduAge | | 1183 | 38.22 | 0.02 | (-64.51, 137.28) | BF_01_=26.16 | BF_01_=13.31 | BF_01_=39.37 |
| wFA | ROSLA | | 1184 | 0.00 | -0.02 | (0, 0) | BF_01_=32.68 | BF_01_=16.40 | BF_01_=48.70 |
| wFA | EduAge | | 1184 | 0.00 | 0.06 | (0, 0) | BF_01_=3.69 | BF_01_=1.87 | BF_01_=5.51 |

***Sup. Table 6 Caption****:* Bayesian analysis of global neuroimaging measures (Y) using a local randomization RD (“ROSLA”). ROSLA using dummy coding TRUE for participants born from September 1957 until Jan 1958 and FALSE for participants born April-August 1957). EduAge is the associational effect of the amount of attained education in years of the same group of participants. The estimate is the median of the posterior reported in raw units. Std. Est is the standardized estimate (for EduAge this is a standardized continuous variable, while ROSLA is a dummy coded variable with 1 corresponding to being impacted by the policy). The estimate, CI & BF are reported for a normal prior (mean = 0, SD = 1). We also report Bayes factors (BF) for other priors. The suffix “_norm” means normalized for head size.
