# Supplementary material for: No effect of additional education on long-term brain structure, a preregistered natural experiment in thousands of individuals
Source: eLife. 2025 Jul 25;13:RP101526. doi: 10.7554/eLife.101526 (PMC12296260; doi:10.7554/eLife.101526)
Supplement: Supplementary file 7. [file elife-101526-supp7.docx]

| Supplementary Table 7: Five Month window Test of Covariates | | | | | | |
| --- | --- | --- | --- | --- | --- | --- |
| **Y** | **X** | **n** | **Estimate** | **Bayes Factor** | **ci_low** | **ci_high** |
| sex | ROSLA | 1972 | -0.04 | BF_01_=12.30 | -0.08 | 0.01 |
| visit_day_correct | ROSLA | 1972 | 107.29 | BF_01_=1.78 | 25.87 | 191.74 |
| visit_day_correct^2^ | ROSLA | 1972 | 384967.33 | BF_01_=3.35 | 57251.82 | 720507.66 |
| headmotion | ROSLA | 1305 | 0.01 | BF_01_=10.67 | 0.00 | 0.03 |
| imaging_center_11026 | ROSLA | 1950 | 0.01 | BF_01_=34.58 | -0.02 | 0.04 |
| imaging_center_11027 | ROSLA | 1950 | 0.02 | BF_01_=28.85 | -0.02 | 0.06 |
| dMRI_25922_1 | ROSLA | 1203 | -0.01 | BF_01_=33.09 | -0.06 | 0.04 |
| dMRI_25921_1 | ROSLA | 1246 | 0.03 | BF_01_=19.13 | -0.02 | 0.08 |
| dMRI_25928_1 | ROSLA | 1208 | 0.07 | BF_01_=1.10 | 0.02 | 0.11 |
| imaging_center_11028 | ROSLA | 1950 | 0.02 | BF_01_=15.85 | -0.01 | 0.04 |
| imaging_center_11025 | ROSLA | 1950 | -0.04 | BF_01_=6.20 | -0.09 | 0.00 |

***Sup. Table 7 Caption****:* Five-month bandwidth Bayesian local randomization analysis of placebo outcomes. Placebo outcomes are a common method to falsify an RD design, by definition, they *should* be unrelated to the natural experiment (ROSLA). ROSLA dummy codes participants born after September 1^st^, 1957. The estimate is the median of the posterior. The estimate, CI & BF are reported for a normal prior (mean = 0, SD = 1).
